# Growth Hormone Effects on Bone Loss-Induced by Mild Traumatic Brain Injury and/or Hind Limb Unloading

**DOI:** 10.1038/s41598-019-55258-9

**Published:** 2019-12-12

**Authors:** Chandrasekhar Kesavan, Nikita M. Bajwa, Heather Watt, Subburaman Mohan

**Affiliations:** 1 Musculoskeletal Disease Center, VA Loma Linda Healthcare System, Loma Linda, CA 92357 USA; 2 Department of Medicine, School of Medicine, Loma Linda University, Loma Linda, CA 92354 USA; 3 Division of Biochemistry, Department of Basic Sciences, School of Medicine, Loma Linda University, Loma Linda, CA 92354 USA; 4 Department of Orthopedics, School of Medicine, Loma Linda University, Loma Linda, CA 92354 USA

**Keywords:** Bone, Osteoporosis

## Abstract

Growth hormone (GH) deficiency and loss of physical activity are common features in traumatic brain injury (TBI) patients that may contribute to bone loss. Therefore, we tested the hypothesis that GH treatment will rescue the hind limb unloading (UL)-induced skeletal deficit in TBI mice. Mild TBI was induced once per day for four consecutive days. UL (right hind limb) and treatment (3 mg/day GH or vehicle) began two weeks after the first TBI episode and lasted for four weeks. GH treatment increased femur BMD and lean body mass but decreased the % fat measured by DXA in the Control group. Micro-CT analysis revealed that the TBI, UL and TBI-UL groups showed reduced tibia trabecular (Tb) bone mass by 15%, 70%, and 75%, respectively compared to Control mice and that GH treatment significantly increased Tb. bone mass in all four groups. Vertebra also showed reduced Tb. bone mass in TBI, UL and TBI-UL groups. GH treatment increased vertebral Tb. bone mass in Control and UL groups but not in the TBI or TBI-UL group. GH treatment increased serum IGF-I levels similarly in TBI, UL and TBI-UL groups at day 14, suggesting the GH effect on liver IGF-I production was unaffected by skeletal UL. In contrast, GH effect on expression of ALP, IGFBP5 and axin2 in bone were compromised by UL. In conclusion, skeletal UL caused a greater Tb. bone deficit than mild TBI alone and that GH anabolic effects in the TBI and UL groups vary depending on the skeletal site.

## Introduction

Mild traumatic brain injury (mTBI) is an injury that occurs after a blow to the head, typically caused by falls, accidents, sports injuries, and military conflicts. Many individuals diagnosed with mTBI are left with significant long-term neurological and physical impairments that have major catabolic effects on many body areas resulting in a poor quality of life. The Department of Defense special report indicates that more than 313,816 service members who served in the Army, Air Force, Navy, and Marines have sustained TBI in training or combat; however, these numbers do not account for unreported cases or those that did not receive medical care^1–5^. These numbers continue to rise annually, with many patients being constrained to bed rest for variable periods of time during acute hospitalization and during subsequent recovery. Physical activity is essential for the continued maintenance of skeletal architectural integrity, therefore, identifying the strategies that may reduce TBI-mediated secondary skeletal effects may improve quality of life and associated disability.

The hypothalamus transmits signals from the brain to the pituitary gland by stimulating endocrine factors that are essential for maintaining homeostasis and regulating skeletal integrity^6^. Many studies have reported hypothalamus-pituitary dysfunction post TBI, with both adverse acute and long-term effects in patients^7–10^. In TBI, the hypothalamus-pituitary function is disrupted, leading to multiple hormone deficiencies, particularly in growth hormone (GH) production. Several studies have reported GH deficiency in 14–21% of TBI patients^7,11^. Growth hormone is a well-known regulator of skeletal homeostasis and is critically involved in regulating normal longitudinal bone growth as well as attainment of peak bone mass^12,13^. We have previously shown that two weeks of GH treatment significantly increased total body bone mineral density (BMD) by as much as 15% in GH deficient *lit/lit* mice^12^. Clinical studies have shown that adults with GH deficiency suffer from low bone turnover osteoporosis, which leads to an increased risk of fracture and mortality^14,15^. GH deficiency is a common adverse effect in TBI patients that may contribute to bone loss. Accordingly, we recently demonstrated that repeated mild TBI to the brain exerts a significant negative effect on bone mass accretion in mice that is caused in part due to alterations of the GH/IGF-1 axis^16^. The effects of mechanical strain on bone is dependent on local actions of IGF-1 expressed by bone cells as conditional disruption of IGF-I in bone cells blocked the mechanical strain anabolic response on bone formation in mice^17^. Based on the findings that TBI leads to dysregulation of the GH/IGF-1 axis and that unloading induces skeletal resistance to IGF-1 effects, we tested if the negative effects of TBI on bone mass are exaggerated by skeletal unloading, and if the negative effects of TBI and unloading on bone mass can be rescued by replacement GH therapy.

## Results

### Mild TBI, UL and GH effect on total body BMD, lean body mass and % fat

**Figure 1:**
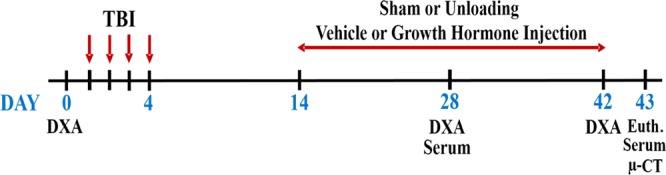
Schematic representation of study timeline.

To test the hypothesis that skeletal unloading exaggerates the negative effects of TBI on trabecular bone mass and that GH treatment ameliorates the negative effects of TBI and/or unloading, mild TBI and sham anesthesia control mice were subjected to unloading (UL) of the right leg and treatment with GH or vehicle as shown in Fig. 1. The body weights of eight groups of mice, i.e., Control-Vehicle (VH), Control-GH, UL-VH, UL-GH, TBI-VH, TBI-GH, TBI-UL-VH and TBI-UL-GH, at different time points are shown in Table 1. DEXA measurements revealed that while total body BMD was not different between Control and TBI groups, unloading significantly reduced total body BMD in both Control and TBI groups (Table 2). GH treatment caused a significant increase in total body BMD at day 42 only in the TBI-UL group. Neither unloading nor mild TBI had a significant effect on total body lean body mass or % fat. However, GH treatment caused a significant increase in lean body mass in all four groups (Control, UL, TBI and TBI-UL) compared to corresponding vehicle treatment. By contrast, % fat was significantly reduced by GH treatment in all four groups (Table 2).

**Table 1:** Body weights in grams of eight groups mice at different time points.

Treatment		VH	GH
Day 0	Control	18.72 ± 0.33	19.48 ± 0.38
	UL	19.04 ± 0.47	18.70 ± 0.59
	TBI	17.92 ± 0.24	18.71 ± 0.30
	TBI-UL	18.67 ± 0.33	19.09 ± 0.29
Day 28	Control	18.68 ± 0.33	21.15 ± 0.34
	UL	18.36 ± 0.43	17.98 ± 0.46
	TBI	18.63 ± 0.25	19.93 ± 0.25
	TBI-UL	18.18 ± 0.34	19.86 ± 0.37
Day 42	Control	19.71 ± 0.26	22.98 ± 0.63
	UL	18.81 ± 0.28	20.23 ± 0.39
	TBI	20.24 ± 0.84	21.88 ± 0.27
	TBI-UL	19.01 ± 0.35	21.06 ± 0.18

**Table 2:** Total body DEXA measurements of the eight groups of mice at different time points.

Treatment	BMD (mg/cm^2^)				Lean Weight (mg)				% Fat			
	Control	UL	TBI	TBI-UL	Control	UL	TBI	TBI-UL	Control	UL	TBI	TBI-UL
***Day 0***												
VH	62.51 ± 1.03	63.77 ± 1.07	62.64 ± 1.20	61.75 ± 0.64	10.67 ± 0.21	10.38 ± 0.33	9.80 ± 0.31^ad^	10.76 ± 0.25^c^	37.76 ± 1.93	39.84 ± 1.01	40.70 ± 1.83	37.56 ± 1.07
GH	63.90 ± 1.54	65.49 ± 0.67^d^	63.04 ± 0.86	61.68 ± 0.41^b^	10.41 ± 0.45	9.58 ± 0.27^acd^	10.54 ± 0.17^b*^	11.31 ± 0.16^b^	41.00 ± 2.65	43.36 ± 1.75^cd^	37.62 ± 1.36^b*^	35.50 ± 0.53 ^ab^
***Day 28***												
VH	65.86 ± 2.07	66.65 ± 2.51	65.59 ± 0.59	65.77 ± 1.94	10.15 ± 0.33	11.12 ± 0.25	10.27 ± 0.23	10.66 ± 0.33	42.06 ± 1.70	31.35 ± 1.17^ac^	39.79 ± 0.93^bd^	33.44 ± 1.01^ac^
GH	67.91 ± 1.40^*^	67.43 ± 2.39	67.12 ± 1.42	66.25 ± 1.91	12.63 ± 0.43^*^	12.19 ± 0.46^ad*^	12.12 ± 0.29^*^	12.93 ± 0.29^b*^	36.56 ± 1.26^*^	32.24 ± 1.00^ac^	36.24 ± 1.14^bd*^	30.06 ± 0.55^ac*^
***Day 42***												
VH	66.31 ± 0.92	63.38 ± 1.55 ^c^	67.72 ± 1.04^bd^	62.59 ± 0.71 ^ac^	10.62 ± 0.28	10.45 ± 0.34	10.91 ± 0.23	11.17 ± 0.18	40.16 ± 1.36	37.57 ± 1.79	36.41 ± 1.16	34.53 ± 1.51
GH	68.63 ± 0.73	66.72 ± 0.97	66.84 ± 0.46	66.55 ± 0.59 ^a*^	13.53 ± 0.29^*^	12.48 ± 0.35^ad*^	13.18 ± 0.21^*^	13.35 ± 0.19 ^b*^	34.19 ± 0.78^*^	31.62 ± 0.95 ^a*^	33.06 ± 0.94^d*^	29.47 ± 0.69^ac*^

^a^*p* < 0.05 vs Control for corresponding treatment and time point; ^b^*p* < 0.05 vs UL for corresponding treatment and time point; ^c^*p* < 0.05 vs TBI for corresponding treatment and time point; ^d^*p* < 0.05 vs TBI-UL for corresponding treatment and time point; *p < 0.05 for corresponding VH treatment.

### Mild TBI, UL and GH effect on femur and tibia BMD

**Figure 2:**
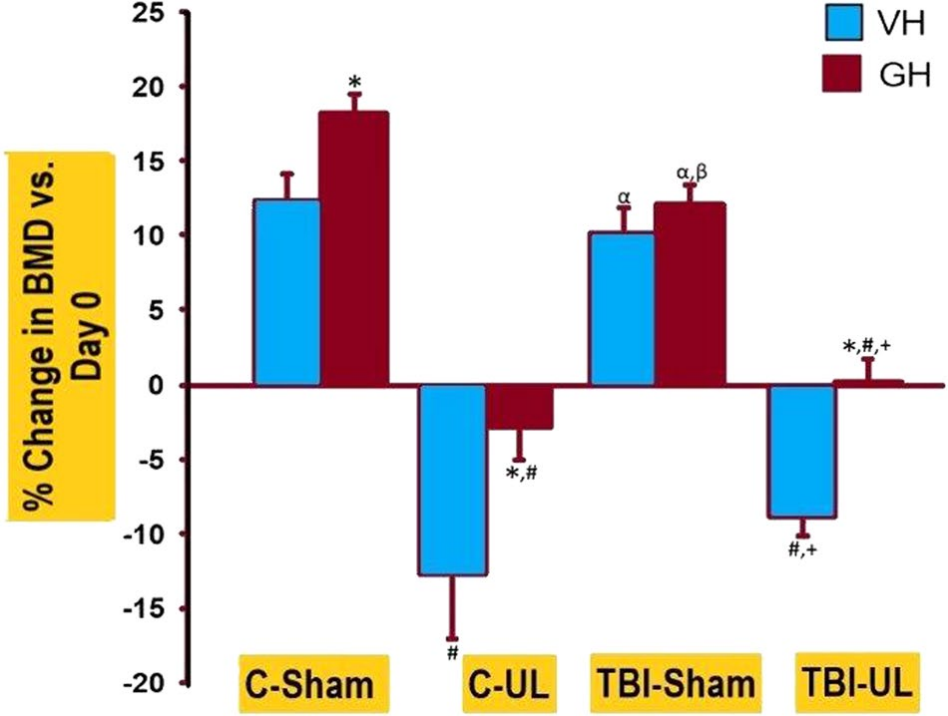
Femoral and Tibial BMD changes in different groups of mice. Th**e** percentage change vs. Day 0 in (A) femoral BMD and (B) tibial BMD at Day 42. *vs. VH; ^#^vs. Control; ^α^vs. UL; ^+^vs. TBI; ^β^vs. Control. All significant levels at *p* < 0.05.

Femur and tibia areal BMD (aBMD) measured by DEXA was not significantly different between the TBI and Control group (Table 3). However, in response to unloading, the femur aBMD was reduced 18–20% in the TBI-UL and UL groups vs. the loaded group (TBI or Control) at day 28 and this reduction remained at day 42. In contrast to the femur, tibia aBMD reduced by only 10–14% in the TBI-UL and UL groups at day 28 and at day 42, this reduction was only 3–5%. GH treatment increased femur and tibia aBMD by 4–12% in the Control, TBI-UL and UL groups but not in the TBI group (Fig. 2).

**Table 3:** DEXA measurements of femur and tibia bone mineral density (BMD) of the eight groups of mice at different time points.

	Treatment	Femur BMD (mg/cm^2^)				Tibia BMD (mg/cm^2^)			
		Control	UL	TBI	TBI-UL	Control	UL	TBI	TBI-UL
	***Day 0***								
LEFT	VH	84.70 ± 1.69	85.72 ± 1.53	84.72 ± 1.67	84.74 ± 1.21	62.73 ± 1.01	62.61 ± 0.97	63.23 ± 1.39	62.76 ± 1.25
	GH	85.38 ± 0.94	86.69 ± 1.39	85.22 ± 1.35	84.77 ± 0.86	63.81 ± 1.41	66.85 ± 1.02 ^cd*^	62.53 ± 1.21^b^	59.86 ± 1.22^ab^
RIGHT	VH	85.65 ± 1.26	88.84 ± 2.12	85.69 ± 1.02	86.18 ± 1.38	65.54 ± 1.04	65.97 ± 1.21	65.75 ± 0.64	65.61 ± 0.75
	GH	88.15 ± 0.78	89.69 ± 1.34 ^cd^	84.90 ± 0.73^ab^	86.52 ± 0.98^b^	66.41 ± 0.48	66.05 ± 1.16	65.17 ± 0.98	64.62 ± 1.00
	***Day 28***								
LEFT	VH	90.57 ± 1.22	84.24 ± 1.63^acd^	92.15 ± 0.94^b^	84.29 ± 1.29^ac^	69.32 ± 0.78	69.47 ± 1.66	71.49 ± 0.92	69.47 ± 1.58
	GH	96.46 ± 1.37^*^	87.66 ± 1.51^ac^	92.51 ± 1.25^b^	88.93 ± 1.69^a*^	72.30 ± 1.37	70.03 ± 1.21	69.85 ± 0.83	68.37 ± 0.88
RIGHT	VH	97.79 ± 1.23	80.68 ± 1.62^ac^	98.53 ± 1.02^bd^	81.15 ± 1.30^ac^	72.63 ± 1.55	65.65 ± 1.10^ac^	74.75 ± 0.71^bd^	64.06 ± 1.33^ac^
	GH	101.64 ± 1.06^*^	85.18 ± 2.11^ac^	98.16 ± 1.28^bd^	86.51 ± 1.22^ac*^	74.39 ± 0.88	67.67 ± 1.30^acd^	71.71 ± 0.92^b*^	73.07 ± 1.81^b*^
	***Day 42***								
LEFT	VH	92.26 ± 1.61	86.87 ± 3.04^c^	94.37 ± 2.03^bd^	85.85 ± 1.69^c^	65.13 ± 1.16	63.48 ± 1.67	68.13 ± 0.92	65.40 ± 1.43
	GH	99.52 ± 1.27^*^	90.84 ± 1.36^ac^	94.83 ± 0.69^abd^	91.50 ± 1.54^ac*^	73.52 ± 2.23^*^	68.50 ± 2.28	69.03 ± 0.46	68.48 ± 1.20
RIGHT	VH	95.81 ± 1.70	77.47 ± 3.86^ac^	94.44 ± 1.43^bd^	78.56 ± 1.09^ac^	68.08 ± 2.14	72.18 ± 2.93	68.09 ± 0.80	66.50 ± 1.91
	GH	100.70 ± 1.11^*^	87.05 ± 1.86^ac*^	95.41 ± 1.06^abd^	86.70 ± 1.26^ac*^	71.71 ± 1.49	71.65 ± 1.65	66.83 ± 1.27^d^	75.84 ± 2.12^c*^

^a^*p* < 0.05 vs Control for corresponding treatment, time point and leg; ^b^*p* < 0.05 vs UL for corresponding treatment, time point and leg; ^c^*p* < 0.05 vs TBI for corresponding treatment, time point and leg; ^d^*p* < 0.05 vs TBI-UL for corresponding treatment, time point and leg; *p < 0.05 for corresponding VH treatment.

### Mild TBI, UL and GH effect on trabecular parameters at the secondary spongiosa of the tibia

**Figure 3:**
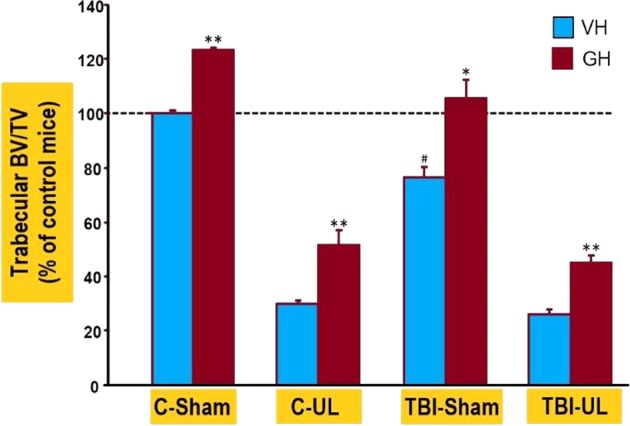
Micro-CT measurements of the trabecular bone measured at the metaphyseal region of the tibia for the different groups of mice. Values are expressed as % of Control-VH group. *p < 0.05; **p < 0.01; ^#^Control-VH vs. TBI-VH, *p* < 0.01.

The TBI mice showed a 22% reduction in trabecular BV/TV at the secondary spongiosa of the tibia when compared to Control (non-impacted mice) group 8 weeks post impact (Fig. 3). The BV/TV phenotype at the secondary spongiosa of the trabecular site was reduced by 70% and 75%, respectively, in the UL and TBI-UL groups (Table 4). A low dose GH treatment nearly rescued the trabecular BV/TV phenotype at the secondary spongiosa of the TBI group. However, GH treatment only partially rescued the trabecular BV/TV phenotype in the TBI-UL and UL compared to GH treated Control group (Table 4).

**Table 4:** Micro-CT measurements of trabecular bone parameters at the secondary spongiosa of the tibia of the eight groups of mice at 8 weeks post-impact.

			VH	GH
Bone Parameters	**BV/TV**	Control	0.19 ± 0.009	0.26 ± 0.012^*^
		UL	0.06 ± 0.012^ac^	0.11 ± 0.012^ac*#^
		TBI	0.15 ± 0.009^abd^	0.23 ± 0.012^bd*#^
		TBI-UL	0.05 ± 0.019^ac^	0.10 ± 0.011^ac*#^
	**Tb. N mm**^**−1**^	Control	5.22 ± 0.13	6.31 ± 0.19^*^
		UL	4.01 ± 0.19^ac^	4.63 ± 0.18^ac*#^
		TBI	4.89 ± 0.14^bd^	6.00 ± 0.18^bd*#^
		TBI-UL	3.75 ± 0.18^ac^	4.57 ± 0.16^ac*#^
	**Tb. Th µm**	Control	0.051 ± 0.001	0.054 ± 0.001
		UL	0.043 ± 0.001^ac^	0.045 ± 0.001^acd#^
		TBI	0.048 ± 0.001^abd^	0.050 ± 0.001^abd^
		TBI-UL	0.042 ± 0.001^ac^	0.042 ± 0.001^abc#^
	**Tb. Sp. µm**	Control	0.19 ± 0.006	0.15 ± 0.008^*^
		UL	0.25 ± 0.008^acd^	0.22 ± 0.008^ac*#^
		TBI	0.21 ± 0.006^bd^	0.16 ± 0.008^bd*#^
		TBI-UL	0.27 ± 0.008^abc^	0.22 ± 0.007^ac#^

^a^*p* < 0.05 vs Control for corresponding treatment; ^b^*p* < 0.05 vs UL for corresponding treatment; ^c^*p* < 0.05 vs TBI for corresponding treatment; ^d^*p* < 0.05 vs TBI-UL for corresponding treatment; *p < 0.05 for corresponding VH treatment. ^#^*p* < 0.05 for GH treatment vs Control-VH.

### Mild TBI, UL and GH effect on bone formation and resorption parameters

The TBI group (impacted mice) showed a 28% (*p* = *0.08*) and 11% (*p* = *0.06*) reduction in bone formation rate (BFR) and mineral apposition rate (MAR) compared to the Control group (non-impacted control mice). In response to unloading, BFR was reduced by 55% in the Control group and 62% in the TBI group (Table 5). The reduction in the BFR in the TBI-UL-VH group was significantly greater than that of UL-VH group. GH treatment increased BFR with a marginal significance (*p* = *0.05*) in the TBI group but not in the Control group. By contrast, GH treatment had no effect on the MAR in the TBI or Control groups. GH treatment increased BFR and MAR similarly in the TBI-UL and UL groups.

**Table 5:** Histomorphometric analysis of bone formation and resorption parameters at the metaphysis of femur of different groups of mice 8weeks post impact.

		VH	GH
**BFR** (BFR mm^2^ × 10^−3^/day)	Control	6.10 ± 0.79	4.0 ± 0.55^*^
	UL	2.71 ± 0.21^ad^	4.20 ± 0.62^c^
	TBI	4.15 ± 0.57^d^	6.35 ± 0.79^abd*^
	TBI-UL	1.59 ± 0.17^abc^	4.24 ± 0.54^c*^
**MAR** (µm/day)	Control	1.25 ± 0.08	1.19 ± 0.04
	UL	1.29 ± 0.04^cd^	1.43 ± 0.04^*^
	TBI	1.05 ± 0.07^b^	1.15 ± 0.07
	TBI-UL	1.09 ± 0.05^b^	1.32 ± 0.1
**TRAP** (% labeled surface)	Control	7.0 ± 1.33	10.42 ± 1.31
	UL	15.43 ± 1.23^ac^	7.70 ± 1.56^*^
	TBI	10.45 ± 1.17^b^	12.34 ± 0.91^#^
	TBI-UL	17.38 ± 1.97^a^	11.63 ± 0.72^#^

^a^*p* < 0.05 vs Control for corresponding treatment; ^b^*p* < 0.05 vs UL for corresponding treatment; ^c^*p* < 0.05 vs TBI for corresponding treatment; ^d^*p* < 0.05 vs TBI-UL for corresponding treatment; *p < 0.05 for corresponding VH treatment. ^#^*p* < 0.05 for GH treatment vs Control-VH.

In addition to bone formation parameters, we also evaluated resorbing surface by measuring the TRAP stained bone surface. The TBI group showed a 49% (*p* = *0.08*) increase in TRAP stained surface reflecting an increase in bone resorption compared to the Control group (Table 5). In response to unloading the TRAP stained surface was increased by 66% and 120% in the TBI-UL (*p* = *0.09*) and UL groups compared to TBI and Control groups, respectively. GH treatment did not alter the TRAP stained resorbing surface in the TBI group but increased it in the Control group with marginal significance (*p* = *0.09*). GH treatment reduced TRAP stained surface in the UL (*p* < *0.01*) and TBI-UL (*p* = *0.08*) groups compared to the corresponding vehicle treated groups.

### Mild TBI, UL and GH effects on cortical bone parameters at the mid-diaphysis of tibia

Micro-CT analysis revealed no significant difference in cortical thickness and apparent BMD in the cortical bone of tibia between TBI and Control groups. GH treatment did not significantly increase cortical thickness or apparent BMD in either Control or TBI group (Table 6).

**Table 6:** Micro-CT measurements of cortical bone parameters at the tibia mid-diaphysis of different groups of mice 8 weeks post impact.

			VH	GH
Bone Parameters	Ct.Th	Control	0.20 ± 0.003	0.21 ± 0.003
		UL	0.15 ± 0.003^ac^	0.18 ± 0.01^acd*#^
		TBI	0.20 ± 0.004^bd^	0.20 ± 0.004^bd^
		TBI-UL	0.14 ± 0.007^ac^	0.16 ± 0.005^abc#^
	Apparent Density	Control	1083 ± 5.72	1074 ± 6.1
		UL	984 ± 10^ac^	994 ± 14^ac#^
		TBI	1085 ± 4.8^bd^	1115 ± 5.0^bd^
		TBI-UL	1006 ± 7.2^ac^	1005 ± 12^ac#^

^a^*p* < 0.05 vs Control for corresponding treatment; ^b^*p* < 0.05 vs UL for corresponding treatment; ^c^*p* < 0.05 vs TBI for corresponding treatment; ^d^*p* < 0.05 vs TBI-UL for corresponding treatment; *p < 0.05 for corresponding VH treatment. ^#^*p* < 0.05 for GH treatment vs Control-VH.

Cortical thickness and apparent BMD at the tibial mid-diaphysis were decreased by 25–30% and 7–9%, respectively, in the TBI-UL and UL groups versus TBI and Control groups. GH treatment increased cortical thickness by 20% *(p* = *0.002*) and 14% (*p* = *0.09*), respectively, in the UL and TBI-UL groups compared to corresponding vehicle treatments. In contrast, GH treatment did not increase apparent BMD in any of the groups (Table 6).

### Mild TBI, UL and GH effects on trabecular parameters of the lumbar vertebra

Micro-CT analysis revealed a 20% (p < 0.01) reduction in trabecular BV/TV at the lumbar vertebra 5 (L5) in the TBI group compared to the Control group (Table 7). Interestingly, the trabecular BV/TV of L5 was reduced by 28% (*p* < *0.01*) in response to long bone unloading in the TBI-UL and UL groups compared to the corresponding control groups. GH treatment significantly increased L5 trabecular BV/TV in Control group by 16%. Additionally, GH treatment caused partial rescue of L5 BV/TV phenotype in the TBI-UL and UL groups but not in the mTBI mice.

**Table 7:** Micro-CT measurements of trabecular bone parameters for the lumbar 5 vertebra of different groups of mice 8 weeks post impact.

			VH	GH
Bone Parameters	BV/TV	Control	0.38 ± 0.019	0.44 ± 0.017^*^
		UL	0.32 ± 0.017^ad^	0.37 ± 0.017^acd^
		TBI	0.31 ± 0.018 ^ad^	0.30 ± 0.017^ab#^
		TBI-UL	0.23 ± 0.018 ^abc^	0.27 ± 0.017^ab#^
	Tb. N mm^−1^	Control	4.91 ± 0.13	5.08 ± 0.12
		UL	4.46 ± 0.12^ac^	4.84 ± 0.12^*^
		TBI	4.99 ± 0.13^bd^	5.02 ± 0.12^d^
		TBI-UL	4.47 ± 0.13^ac^	4.61 ± 0.13^ac^
	Tb. Th µm	Control	0.062 ± 0.001	0.061 ± 0.001
		UL	0.052 ± 0.001^ac^	0.053 ± 0.001^ac#^
		TBI	0.061 ± 0.001^bd^	0.059 ± 0.001^bd^
		TBI-UL	0.051 ± 0.001^ac^	0.055 ± 0.001^ac*#^
	Tb. Sp. µm	Control	0.21 ± 0.006	0.20 ± 0.006
		UL	0.22 ± 0.006^ac^	0.21 ± 0.006^*^
		TBI	0.20 ± 0.006^bd^	0.20 ± 0.006
		TBI-UL	0.22 ± 0.006^c^	0.22 ± 0.006^a^

^a^*p* < 0.05 vs Control for corresponding treatment; ^b^*p* < 0.05 vs UL for corresponding treatment; ^c^*p* < 0.05 vs TBI for corresponding treatment; ^d^*p* < 0.05 vs TBI-UL for corresponding treatment; *p < 0.05 for corresponding VH treatment. ^#^*p* < 0.05 for GH treatment vs Control-VH.

### Mild TBI, UL and GH effect on serum IGF-I and ALP levels

There was a modest 6% decrease in serum IGF-I levels at day 14 and day 43 in the TBI mice compared to Control but this difference did not achieve statistical significance. GH treatment increased serum IGF-I levels similarly in the four groups at day 14 (Table 8). In addition to IGF-I, we also evaluated circulating ALP levels in all groups of mice at day 14 and 43. The twenty percent reduction in serum ALP in the TBI group compared to Control group at day 14 was not significant (*p* = *0.20*). GH treatment increased serum ALP in the Control but not TBI mice (Table 9). Serum ALP was reduced by 42% at both time points in the vehicle treated TBI-UL group compared to TBI group while it was reduced by 39% in the UL group compared to Control group at day 14 (Table 9). GH treatment increased ALP levels by 70% (*p* = *0.001*) and 100% (*p* = *0.001*) at day 14 in the TBI-UL group and in the UL group, respectively.

**Table 8:** Serum levels of IGF-1 at day 14 and 43 in different groups of mice.

	IGF-1 (ng/ml)			
	Control		TBI	
	Control	UL	TBI	TBI-UL
***Day 14***				
VH	371.25 ± 28.46	277.67 ± 23.24^acd^	330.79 ± 26.35^b^	311.81 ± 24.65^ab^
GH	497.33 ± 23.24^*^	439.10 ± 22.05^*#^	425.78 ± 23.24^*^	426.59 ± 21.02^*^
***Day 43***				
VH	536.94 ± 25.22	428.63 ± 21.99^ac^	509.50 ± 17.99^bd^	428.69 ± 12.02^ac^
GH	599.56 ± 51.39	514.50 ± 47.51	610.05 ± 36.09^*^	549.40 ± 44.89^*^

^a^*p* < 0.05 vs Control for corresponding treatment; ^b^*p* < 0.05 vs UL for corresponding treatment; ^c^*p* < 0.05 vs TBI for corresponding treatment; ^d^*p* < 0.05 vs TBI-UL for corresponding treatment; *p < 0.05 for corresponding VH treatment. ^#^*p* < 0.05 for GH treatment vs Control-VH.

**Table 9:** Serum ALP activity at day 14 and 43 for the different groups of mice.

Treatment	ALP Activity (Arbitrary units)			
	Control		TBI	
	Control	UL	TBI	TBI-UL
***Day 14***				
VH	0.92 ± 0.09	0.56 ± 0.08^a^	0.74 ± 0.04^d^	0.43 ± 0.06^ac^
GH	1.20 ± 0.17^*^	1.0 ± 0.11^*^	0.79 ± 0.05^a^	0.75 ± 0.08^a^
***Day 43***				
VH	0.94 ± 0.08	0.98 ± 0.16^d^	0.97 ± 0.14^d^	0.56 ± 0.06^abc^
GH	1.10 ± 0.12	0.88 ± 0.13	1.0 ± 0.019^d^	0.59 ± 0.07^ac#^

^a^*p* < 0.05 vs Control-VH; ^b^*p* < 0.05 vs UL for corresponding treatment; ^c^*p* < 0.05 vs TBI for corresponding treatment; ^d^*p* < 0.05 vs TBI-UL for corresponding treatment; *p < 0.05 for corresponding VH treatment. ^#^*p* < 0.05 for GH treatment vs Control-VH.

**Figure 4:**
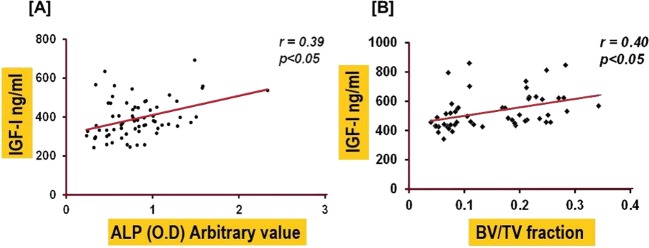
Correlation analysis between circulating IGF-I versus ALP at day 14 (**A**) and IGF-I versus BV/TV at day 43 (**B**).

Pearson correlation analysis revealed a positive association between serum IGF-I and ALP measured at day 14 (*r* = *0.39, p* = *0.001*) and day 43 (r = *0.29, p* = *0.02*) and with the BV/TV phenotype for ALP measured at day 43 (r = 0.37, *p* < *0.05*) and for IGF-I measured at day 43 (r = 0.40. *p* < *0.05*). Correlation plots for IGF-I vs ALP at day 14 and IGF-I vs tibia trabecular BV/TV at day 43 are shown in Fig. 4.

### Gene expression changes

**Figure 5:**
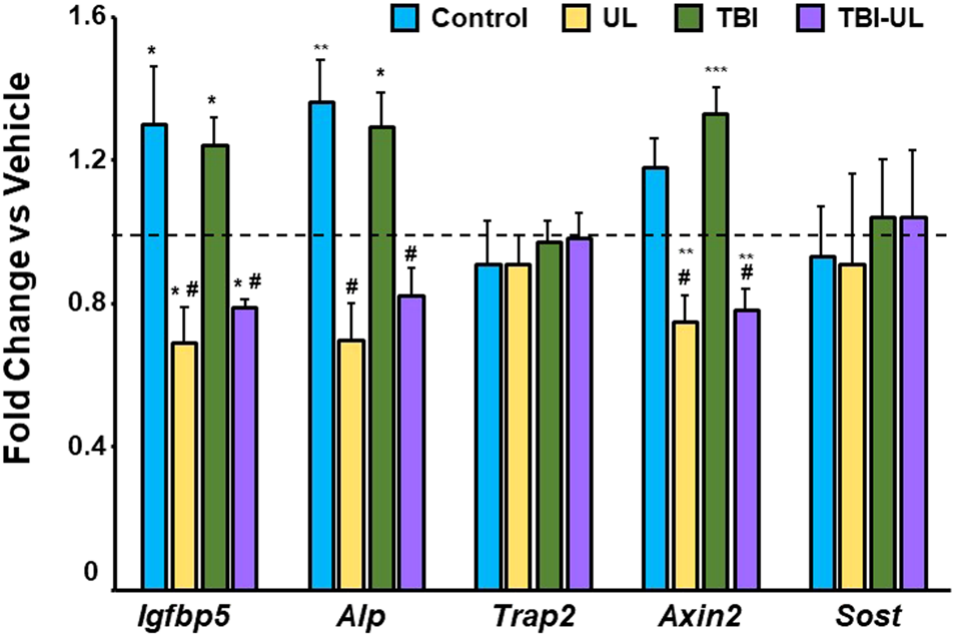
GH-induced gene expression changes in the TBI and/or unloaded mice. Values are expressed as fold change of corresponding vehicle treatment. *p < 0.1; **p < 0.05; ***P < 0.01 vs vehicle; ^#^P < 0.01 vs GH treatment in the loaded group (n = 6 for all groups except for control vehicle, n = 5).

Figure 5 shows GH-induced changes in expression levels of GH target (*Igfbp5*), Wnt signaling targets (*Axin2*, *Sost*) and bone markers (*Alp, Trap2*). GH treatment increased expression levels of *Igfbp5, Alp* and *Axin2* in the loaded but not unloaded bones of control and TBI mice. The expression levels of *Sost* and *Trap2* were unaffected after 24 hour GH treatment. Furthermore, GH-induced increase in expression levels of *Alp* correlated positively with changes in *Axin2* mRNA levels in the unloaded and loaded bones of control and TBI mice (*r* = *0.55, P* < *0.01*).

## Discussion

In this study, we demonstrate that repeated mild TBI exerts significant negative effects on trabecular bone volume at multiple skeletal sites, i.e. tibia and lumbar vertebra, 6 weeks post-impact. In contrast to trabecular bone parameters, repeated mild TBI did not exert significant effects on the cortical bone parameters. The lack of TBI effects on the cortical bone is consistent with what was found in an earlier study^16^. There are a number of potential explanations for differential effects of TBI on cortical versus trabecular bone: First, it is estimated that 80% of bone remodeling activity takes place in cancellous bone, although cancellous bone only comprises 20% of bone^18^. Thus, trabecular bone is likely to be more susceptible to changes induced by physiological and pathological perturbations. Second, it is known that mechanisms regulating cortical and trabecular bone formation are different. Cortical bone is primarily formed by osteoblasts that are derived by direct differentiation of mesenchymal stem cells. By contrast, trabecular bone is derived via endochondral ossification in which chondrocyte produced cartilage is replaced by bone. In this regard, there is now evidence that some of the chondrocytes transdifferentiate into bone matrix producing osteoblasts during endochondral bone repair and secondary ossification of the epiphyses^19,20^. Third, the neuroendocrine signals impacted by repeated mild TBI may affect trabecular bone formation and/or remodeling and not cortical bone.

A key aspect of this study was to determine if the negative effects of repeated mild TBI on the skeleton were exaggerated by unloading of the skeleton. The rationale to investigate the combined effects of TBI and skeletal unloading on bone metabolism was based on the establishment that many individuals with TBI are immobilized for considerable lengths of time and that the GH/IGF axis, a key hypothalamic-pituitary axis impacted by TBI, is critically involved in regulating bone metabolism changes in response to both unloading and TBI^21^. To study the effects of unloading, we adapted a unilateral hind limb unloading method that was previously shown to reduce muscle mass by 20% after 7 days of unloading^22^. In the unilateral unloading model, the right leg was subjected to unloading and the contralateral left leg was used as a control to more reliably evaluate changes in response to local unloading. Four weeks of unloading caused a dramatic 68% decrease in trabecular bone mass in the tibia of Control mice, thus demonstrating the effectiveness of the unloading model used. In the TBI-UL mice, the trabecular bone mass was reduced by 74% compared to Control mice. While we anticipated that the combination of TBI and skeletal unloading would cause a greater reduction in trabecular bone mass compared to either treatment alone, the trabecular BV/TV in the TBI-UL-VH was not significantly different from that of UL-VH group. One potential explanation for the lack of a combination effect could be due to the severity of the unloading method used. Further time course studies using partial skeletal unloading are necessary to fully evaluate if unloading exaggerates the TBI effects on the skeleton.

Surprisingly, local hindlimb unloading also caused a significant reduction in the trabecular bone mass of lumbar vertebra of Control mice. However, the magnitude of reduction of trabecular bone mass at a distal lumbar site was much less (16%) compared to the unloaded tibial trabecular bone mass (68%). The mechanism by which the unilateral right hind limb unloading causes trabecular bone changes in L5 remains to be established. One potential explanation is that local unloading may induce systemic changes in circulating osteogenic growth factors (e.g. IGF-I) that could contribute to reduced trabecular bone volume in the vertebra. In any case, it was interesting that the trabecular bone mass deficit of 40% in the TBI-UL compared to Control mice was significantly greater that seen in the UL (16%) and TBI (18%) mice, thus suggesting that skeletal unloading did indeed exaggerate the TBI effects at least in the trabecular bone phenotype of the L5 vertebra. Skeletal unloading also caused a 25–30% reduction in cortical thickness and 7–9% reduction in apparent BMD in UL and TBI-UL groups. The TBI-UL group showed an additional 7% (*p* = *0.20*) cortical thickness reduction in the tibia compared to UL group suggesting that the negative effect of mTBI on cortical bone mass may become evident only when it is challenged for example with immobilization. Further studies are needed to examine the mechanism for the negative effect of TBI and UL on the cortical bone mass deficit seen in the tibia.

There is now extensive data in the literature to demonstrate that disruption of GH-IGF-axis via the hypothalamic-pituitary-axis (HPA) in response to trauma has a significant negative effect on bone^16,23^. In addition, we and others have shown that the mechanical strain effect on bone formation is dependent on IGF actions^17,24–26^. Based on these findings, we predicted that GH therapy would ameliorate some of the negative effects of TBI and/or unloading on the skeleton. Our findings that GH treatment for four weeks significantly increased lean body mass and reduced the percentage total fat in all four study groups suggest that the dose of GH used was effective. Accordingly, our study showed for the first time that a low dose treatment with GH treatment rescued the trabecular BV/TV phenotype in the TBI group similar to the Control group. However, GH treatment only partially rescued the trabecular bone volume deficit in the UL and TBI-UL groups. In this regard, there is evidence in the literature that skeletal unloading induces resistance to IGF-I. A previous study demonstrated that IGF-I treatment increased bone formation, as expected, in control animals but failed to stimulate bone formation in the hindlimb elevated unloaded animals^27^. In addition, it was found that IGF-I failed to activate the IGF-I receptor and downstream signaling cascade to promote proliferation in osteoprogenitors isolated from unloaded bones^28^. In terms of mechanisms for IGF-I resistance caused by skeletal unloading, it has been found that unloading disrupts integrin regulation of IGF-I but not PDGF signaling in osteoblasts^28^. The issue of whether the impaired GH response in TBI-UL mice is caused by disruption of integrin-mediated IGF-I signaling in bone cells remains to be determined.

Our histomorphometric studies reveal that both reduction in trabecular bone formation and increased bone resorption contributed to the dramatic reduction in trabecular bone mass of the tibia of hindlimb unloaded mice compared to control mice. Similarly, TBI-induced reduction in trabecular bone mass is also caused by both a reduction in bone formation and increased resorption. GH treatment increased both bone formation and resorption in the UL, TBI and TBI-UL groups. These data are consistent with findings from human clinical studies that show elevated levels of both bone formation and resorption parameters in GH treated GH deficient adults^29^. Furthermore, IGF-I has been shown to promote osteoclast differentiation^30^. In our study, we failed to detect an increased bone formation response in GH treated Control mice although trabecular bone mass was significantly increased compared to vehicle treated Sham mice. However, bone resorption as measured by the percentage of TRAP labeled surface was increased in GH treated control mice. One potential explanation for a lack of change in the bone formation rate in the GH treated Control mice could be due to the fact that we might have missed the anabolic effect of GH in this group since calcein was administered at the end of the study for histomorphometric analyses. It may be necessary to administer calcein earlier to see if the GH anabolic effect on bone is restricted during the early treatment period in the Control mice.

In a previous study^16,31^, we demonstrated that serum IGF-I levels were reduced in TBI mice, thus suggesting involvement of the GH/IGF axis in mediating the negative effects of TBI on trabecular bone formation. While the reduced serum IGF-I levels in TBI mice were not significant, we found that serum IGF-I levels showed significant positive correlation with both serum levels of ALP, a bone formation marker, as well as trabecular bone volume. While these data are suggestive of a role for GH/IGF axis in mediating TBI and unloading effects, further studies are needed to establish a cause and effect relationship between changes in serum IGF-I levels and trabecular bone volume.

Our data show that the GH anabolic effects in TBI and UL mice vary depending on the skeletal site. In this regard, it is now well established that TBI as well as unloading induces changes in other signaling pathways besides IGF-I. For example, TBI induces changes in serum levels of other endocrine hormones such as thyroid hormones, ACTH and gonadotropins, besides GH^8–11^. In addition, serum levels of a number of neuropeptides such as substance P, leptin, NPY, CGRP are known to be increased in TBI patients^23^. Similarly, unloading induces changes in a number of signaling pathways such as IGF-I, Wnt, estrogen and leptin that play key roles in regulating bone metabolism^24^. In previous studies, we reported that a single injection of GH increased expression levels of several genes in the Wnt signaling pathway in bone, in addition to the anticipated increase in the expression of GH/IGF axis genes^32^. Based on the findings from our and other laboratories that Wnt signaling is critically involved in mediating skeletal anabolic response to mechanical loading^24,33,34^, we evaluated expression levels of Wnt targets in response to GH treatment in the unloaded and loaded bones of control and TBI mice. Our findings (Fig. 5) show that GH response in the expression levels of bone formation marker (ALP), GH target (IGFBP-5) and Wnt target (axin2) were reduced in the unloaded bones compared to loaded bones in both control and TBI mice. Furthermore, the changes in expression levels of ALP correlated with changes in the expression levels of axin2 (*r* = *0.55*), thus suggesting that diminished GH response in the unloaded bones of TBI and control mice could in part be due to reduced Wnt signaling. Thus, that the skeletal site-specific GH effects in the TBI and/or UL groups could in part be due to differing interactions between changes in GH/IGF-I axis and other osteogenic signaling pathways that are altered by TBI and/or unloading. Further studies are needed to evaluate the mechanisms for unloading induced deficit in the expression of Wnt signaling targets by GH and if the diminished bone formation response to GH in the unloaded bones can be rescued by correcting Wnt signaling deficit.

In conclusion, our findings demonstrate that repetitive mild TBI along with hind limb unloading caused a severe skeletal deficit in mice and that GH treatment partially rescued trabecular bone deficit caused by the combined effects of TBI and unloading. These data demonstrate the potential usefulness of low dose GH therapy to ameliorate bone loss in TBI patients who are bedridden.

## Methods

### Animals

Nine-week old female C57BL/6J mice (n = 81) were purchased from Jackson Laboratory (Bar Harbor, Maine). All the animals were housed under standard conditions with 14-hours of light and 10-hours of darkness, with unrestricted access to food and water in the Veterinary Medical Unit in the VA Loma Linda Healthcare System. Mice were allowed to acclimate for one week before experiments were initiated. The animals were randomly assigned to the following groups: Control-Vehicle (VH; n = 9), Control-GH (n = 10), UL-VH (n = 10), UL-GH (n = 10), TBI-VH (n = 10), TBI-GH (n = 11), TBI-UL-VH (n = 10), and TBI-UL-GH (n = 11). A schematic representation of the study design is shown in Fig. 1. All experimental protocols were approved by the Institutional Animal Care and Use Committee of the VA Loma Linda Healthcare System. All procedures performed followed the ethical guidelines for animal studies.

### Traumatic brain injury

TBI was induced as previously reported^31,35^. A 75 gram brass weight was used to generate the impact on the skull. To obtain a consistent impact on the mouse skull, a tube was positioned above the mouse calvaria, between the ears. Under isoflurane anesthesia, mice were subjected to repeated mTBI from a height of 1.5 m, once per day, for four consecutive days^31^. The control mice received isoflurane anesthesia only. All mice survived the duration of the experiment and no paralysis or skull fractures were observed.

### Hind limb unloading

Right leg unloading was created with modifications to previous models^36^. In order to prevent injury to the leg and body, staples were not used as described in previous studies. Mice were anesthetized with isoflurane and the right leg was immobilized with a wooden splint and bound with electrical tape, metal wire, and duct tape. The tapes wrapped the entire leg to ensure the knee was inflexible. The tape was reinforced if the mice chewed it off. The pliable metal wire was wrapped in between the layers of tape to deter mice from chewing completely through the dressing. Mice dragged their right leg and used their left leg normally. Control animals received isoflurane anesthesia only. All mice survived the duration of the experiment and no limb loss occurred. Antibiotic ointment was applied to exposed feet and hips to prevent infections.

### Growth hormone treatment

Mice were injected with recombinant human growth hormone (Biosidus, Buenos Aires, Argentina), diluted in phosphate-buffered saline (PBS), or vehicle (PBS) intraperitoneally at a dose of 3 mg/day. Doses were adjusted weekly for body weight. Treatment began two weeks after the first TBI and lasted for four weeks.

### Serum analyses

Blood was collected from mice at baseline and day 14 using a retro-orbital method and immediately after euthanasia at day 42. The serum was collected and stored at −80 °C and used for quantitating IGF-I (R&D system, USA) and osteocalcin levels (ALPCO, USA) with ELISA kits.

### Dual-energy x-ray absorptiometry (DXA)

Bone mineral content (BMC), bone area (BA), bone mineral density (BMD), lean weight (LW), and % fat of mice were measured by dual-energy X-ray absorptiometry (DXA) using the Ultrafocus Faxitron (Faxitron Bioptics LLC., Tucson, AZ) according to published methods^37^. Eighty-one mice were measured at baseline, day 14, and day 28.

### Micro computed tomography (µ-CT)

For *ex-vivo* and *in vivo* µ-CT evaluations, a high-resolution tomography image system (viva CT40; Scano Medical AG, Bruttisellen, Switzerland) was used to measure cortical and trabecular bone parameters, as previously described^37,38^. Routine calibration was performed once per week using a three-point calibration phantom corresponding to a density range from air to cortical bone. The bones were scanned at a resolution of 10 µm with a 55 and 65 kVp X-ray for trabecular and cortical bone. After acquiring the radiographic data, images were reconstructed using the 2-D image software provided by Scano. Every 10 sections of the cortical or trabecular bone were outlined, and the intermediate sections were interpolated with the contouring algorithm to create a volume of interest, followed by the three-dimensional analysis. For cortical bone measurements, 1 mm of cortical bone was scanned 3 mm away from the tibia-fibula junction (TFJ). The TFJ was used as a reference point to minimize variation arising from the bone position between mice. For trabecular bone measurements, the bones were scanned from 0.36 mm proximal to the growth plate. The exact numbers and location of the slices used for the analysis were corrected for bone length such that the analyzed regions were anatomically comparable between samples. Parameters such as bone volume (BV, mm^3^), bone volume fraction (BV/TV), apparent density (mg HA/ccm), trabecular number (Tb. N, mm^−1^), trabecular thickness (Tb. Th, μm) and trabecular space (Tb. Sp, μm) were evaluated in the impacted and non-impacted groups.

### Dynamic calcein labeling and histomorphometry

Mice were intraperitoneally injected with calcein (20 mg/kg) 10 and 4 days before euthanasia. Bone samples were collected and fixed in 10% formalin. Calcein labeling was visualized using the Olympus BX60 fluorescence microscope (Olympus Corporation, Center Valley, PA, USA) and analyzed using the OsteoMeasure software (Osteometrics, Inc., Decatur, GA, USA). Trabecular bone parameters such as mineral surface (MS, mm), bone formation rate (BFR, mm^2^ × 10^−3^/day), and mineral apposition rate (MAR, µm/day) were calculated at the tibial metaphysis (secondary spongiosa) in the control and impacted mice as previously described^39^. Additionally, the bone resorbing surface was also measured by tartrate-resistant acid phosphatase (TRAP) staining at the trabecular site. The measurements were done at 100X magnification.

### Gene expression measurements

Ten-week old C57BL/6J mice were subjected to mild TBI (n = 12) or sham anesthesia (n = 11) and hind limb unloading as described above. One week after hind limb unloading, control and TBI mice were administered with two injections of GH (1.5 mg/kg) or vehicle control, eight hours apart, and euthanized 24 hours after the first injection. Tibia were removed, flushed with cold PBS to remove marrow and metaphyses were frozen in liquid nitrogen immediately for RNA extraction. Total RNA was extracted using RNA extraction kit from Qiagen and used for measurement of gene expression changes by real time RT-PCR as described^17^. The data were normalized using the endogenous control gene (PPIA) and the normalized Ct values were subjected to the 2^−∆∆^Ct formula to calculate the fold change of GH treated versus vehicle treated group.

### Statistical analysis

ANOVA was used to compare differences between the groups for the bone parameters measured by DEXA, micro-CT analysis and for serum analysis. Student t-tests were used to compare histomorphometric analysis data. An alpha level of *p* < 0.05 was considered statistically significant. Data was analyzed using SPSS (version 21) software and presented as the mean ± SEM.
